# Long-Term Follow-Up of Elderly Patients with Acute Myeloid Leukemia Treated with Decitabine: A Real-World Study of the Apulian Hematological Network

**DOI:** 10.3390/cancers14030826

**Published:** 2022-02-06

**Authors:** Michelina Dargenio, Giuseppe Tarantini, Nicola Cascavilla, Enzo Pavone, Pellegrino Musto, Patrizio Mazza, Lorella Melillo, Domenico Pastore, Attilio Guarini, Caterina Buquicchio, Maria Paola Fina, Vincenzo Federico, Teresa Maria Santeramo, Marina Aurora Urbano, Mariangela Leo, Vera Carluccio, Paola Carluccio, Mario Delia, Daniela Carlino, Carolina Vergine, Vito Pier Gagliardi, Giuseppina Greco, Silvia Sibilla, Mariachiara Abbenante, Giovanni Rossi, Giuseppina Spinosa, Annamaria Mazzone, Lara Aprile, Vincenza de Fazio, Crescenza Pasciolla, Giorgina Specchia, Nicola Di Renzo

**Affiliations:** 1 Hematology and Stem Cell Transplant Unit, “Vito Fazzi” Hospital, 73100 Lecce, Italy; miviforina@tiscali.it (M.D.); mariapaola79@libero.it (M.P.F.); federico.ematolecce@gmail.com (V.F.); danielac80@libero.it (D.C.); carolinavergine@libero.it (C.V.); 2 Hematology Unit, Dimiccoli Hospital, 76121 Barletta, Italy; giuseppetarantini0@gmail.com (G.T.); caterinabuquicchio@libero.it (C.B.); taniasanteramo@gmail.com (T.M.S.); mariangela_leo@libero.it (M.L.); carlucciovera@gmail.com (V.C.); 3 Hematology Unit, IRCCS “Casa Sollievo della Sofferenza”, S. Giovanni Rotondo, 71013 Foggia, Italy; nicola.cascavilla@tin.it (N.C.); lmelillo@ospedaliriunitifoggia.it (L.M.); m.abbenante@operapadrepio.it (M.A.); giovannirossi.fr@gmail.com (G.R.); 4 Hematology and Transplant Unit, Cardinal Panico Hospital, 73039 Tricase, Italy; enzopavone@libero.it (E.P.); giuseppina.greco@gmail.com (G.G.); salentoematologia@piafondazionepanico.it (S.S.); 5 Unit of Hematology and Stem Cell Transplantation, AOUC Policlinico, 70124 Bari, Italy; pellegrino.musto@uniba.it (P.M.); paola.carlucciomd@gmail.com (P.C.); mario.delia74@gmail.com (M.D.); vitopier86@gmail.com (V.P.G.); 6 Department of Emergency and Organ Transplantation, University of Bari “Aldo Moro”, 70124 Bari, Italy; 7 Hematology Unit, Department of Hematology-Oncology, Moscati Hospital, 74010 Taranto, Italy; patrizio.mazza@libero.it (P.M.); annamariamazzone1975@gmail.com (A.M.); laraaprile84@gmail.com (L.A.); 8 Hematology Unit, Azienda Ospedaliero Universitaria—Ospedali Riuniti, 71122 Foggia, Italy; gspinosa@ospedaliriunitifoggia.it; 9 Hematology Unit, A. Perrino Hospital, 72100 Brindisi, Italy; domenico.pastore0@gmail.com (D.P.); sa.marina36@gmail.com (M.A.U.); 10 Hematology Unit, Giovanni Paolo II IRCCS Cancer Institute Oncology Hospital, 70124 Bari, Italy; attilioguarini@gmail.com (A.G.); cinziadefazio@yahoo.it (V.d.F.); crescenza.pasciolla@gmail.com (C.P.); 11 School of Medicine, University of Bari “Aldo Moro”, 70124 Bari, Italy; specchia.giorgina@gmail.com

**Keywords:** acute myeloid leukemia, decitabine, elderly, real-world study, treatment

## Abstract

**Simple Summary:**

This Italian real-life study conducted between 2013 and 2021 and including 199 acute myeloid leukemia (AML) patients demonstrates, after a median follow-up of almost 3 years, how decitabine administered to AML patients not suitable for intensive chemotherapy is effective and well tolerated, even in a population of truly elderly patients with frequent comorbidities.

**Abstract:**

Decitabine, a DNA hypomethylating agent, was approved for use in adults with acute myeloid leukemia (AML) not eligible for standard chemotherapy and is now widely accepted as standard treatment. Although a number of clinical trials demonstrated its benefits in elderly AML patients, older adults and patients with frequent comorbidities are typically under-represented in such settings. Thus, the aim of the present study is to evaluate, in a real-world setting, the effectiveness and toxicity of decitabine administered as a single agent in unselected previously untreated elderly AML patients not eligible for intensive chemotherapy. In nine hematological departments of the Apulian Hematological Network (REP), we enrolled 199 patients (median age: 75.4 years; range: 61–91) with de novo (*n* = 94) or secondary/therapy-related (*n* = 105) AML treated with decitabine 20 mg/m^2^ for five days every 4 weeks. Hazard ratios (HR) and their 95% confidence intervals (CI) were estimated using multivariate Cox regression. The average number of cycles administered per patient was 6.3 (SD: 6.0; median: 5 cycles). Complete response was achieved by 31 patients (15.6%) and partial response by 57 (28.6%), for a total of 88 responders overall (44.2%). After a median follow-up of 33.6 months, median OS was 8.7 months (95% CI: 7.4–10.3), and the 6-month, 1-year, and 3-year OS rates were 62.7%, 37.0%, and 7.1%, respectively. Mortality was increased in AML patients with ≥3 comorbidities (HR = 2.45; 95% CI: 1.18–5.08) vs. no comorbidities and in those with adverse karyotype (HR = 1.58; 95% CI: 1.05–2.38) vs. favourable or intermediate profile. Infection was the main registered adverse event (46.0%). In conclusion, this REP real-life study demonstrates, after a follow-up of almost 3 years, how decitabine administered to AML patients not suitable for intensive chemotherapy is effective and well tolerated, even in a population of truly elderly patients with frequent comorbidities.

## 1. Introduction

According to the IARC GLOBOCAN, over 450,000 incident cases of leukemia are diagnosed worldwide, and over 300,000 patients die of this cancer every year [1]. Acute myeloid leukemia (AML), a clonal malignant disorder characterized by an arrest of normal myeloid differentiation and abnormal proliferation of myeloid precursors, resulting in hematopoietic insufficiency, is one of the four main leukemia subcategories, accounting for about 30% of all leukemia cases occurring in adult populations [2,3]. AML is a disease of aging subjects—the median age at diagnosis is about 65 years—with a poor prognosis, particularly in the elderly. In patients aged 65 years or older, the 5-year relative survival rate in the USA is still only 12.5%, although there has been a trend to improvements in survival over time [4]. The latter is, at least in part, explained by the more frequent decision to treat (older) AML patients [4], as well as by an increasing range of available treatment options. Treatment selection for older patients is particularly challenging, and requires a comprehensive consideration of disease-specific characteristics, as well as full evaluations of comorbidities and functional status, which influence treatment tolerance and efficacy [5,6].

Epigenetic therapy with decitabine is a treatment option for a large proportion of elderly AML patients, including those presenting with unfavorable prognostic factors and/or unfit for intensive chemotherapy. Following the positive results of randomized clinical trials [7,8], decitabine use was widely adopted in the last few years. A limited number of investigations from real-life clinical practice—i.e., from studies based on heterogeneous patient settings—have reported information about the toxicity and effectiveness of decitabine for the treatment of AML patients in a real-world context [9,10,11,12,13,14,15]. European data are particularly scanty in this setting [16,17,18].

Herein, we aimed to provide further information about decitabine safety and effectiveness based on a long-term study of unselected AML patients treated with decitabine as frontline therapy. To our knowledge, these are the most mature data on decitabine treatment in unfit patients with AML.

## 2. Materials and Methods

Data were derived from a multicentric, observational study aimed at examining the tolerability and effectiveness of decitabine therapy as a first-line treatment for AML patients in a real-world setting. The study was conducted in a network of nine university or general hospitals of the Apulian Hematological Network (REP), South-Eastern Italy. Patient enrolment started in September 2013, and the current analysis is based on follow-up data collected until March 2021. Each patient was included if: (a) he/she had a diagnosis of AML at the onset; (b) he/she was 65 years or older or was not eligible for intensive chemotherapy according to the GITMO/SIES/SIE guidelines [19], and was receiving decitabine as initial treatment. All patients were treated with decitabine at the recommended daily dose of 20 mg/m^2^ (5 days of treatment every 4 weeks). Patients with acute promyelocytic leukemia (APL) as well as those with central nervous system involvement of AML were excluded. The full study database included 207 patients with AML. After data checking, 8 patients (3.9%) were excluded due to a lack of key information. Thus, this analysis is based on a total of 199 patients with AML, aged 61–91 years (median age, 75 years). The median follow-up duration in this cohort, computed according to Schemper and Smith method, was 33.6 months [20].

Information on patient and disease characteristics, laboratory values at baseline, treatment, and follow-up data were collected and imputed in a standardized format into a Microsoft Excel spreadsheet by the investigators at each center. All cases were categorized as (a) AML with recurrent genetic abnormalities, (b) AML with myelodysplasia-related changes (AML-MRC), (c) AML therapy-related (t-AML), and (d) AML not otherwise specified AML-NOS according to the 2008 WHO classification [21]. The cytogenetic risk of all patients was re-classified according to the 2017 European LeukemiaNet (ELN) risk stratification [5]. Bone marrow assessment was conducted at baseline, after the fourth cycle, and after, whenever clinically indicated. HemaVision -28N screening test for 28 translocations and more than 145 breakpoints associated with leukemia was used for molecular testing. Response to treatment was defined according to the criteria proposed by ELN: (a) complete remission (CR), defined as bone marrow blasts <5%; absence of circulating blasts and blasts with Auer rods; absence of extramedullary disease; ANC ≥ 1.0 × 10^9^/L (1000/mL) and platelet count ≥100 × 10^9^/L (100,000/mL); (b) CR with incomplete hematologic recovery (CRi): all CR criteria except for residual neutropenia (<1.0 × 10^9^/L [1000/mL]) or thrombocytopenia ≤100 × 10^9^/L [100,000/mL]); (c) partial remission (PR): all hematologic criteria of CR; decrease of bone marrow blast percentage to 5% to 25%; and decrease of pretreatment bone marrow blast percentage by at least 50%; (d) progressive disease (PD) defined as >50% increase in marrow blasts over baseline or persistent marrow blast percentage of >70% over at least 3 months, without at least a 100% improvement in ANC to an absolute level (>0.5 × 10^9^/L [500/mL], and/or platelet count to >50 × 10^9^/L [50,000/mL] non transfused); >50% increase in peripheral blasts and appearance of extramedullary disease. Patients with stable or progressive disease were defined as non-responders. The safety profile was assessed by adverse events, medical histories, physical examinations, concurrent medications, and central laboratory assessments. Toxicities were graded according to National Cancer Institute Common Toxicity Criteria, version 4.3. All data entered in the spreadsheet were fully anonymized. Written informed consent was obtained from all living patients. For deceased patients, the treatment of personal data was compliant with the provisions of the GDPR n.679/2016. The study was conducted in accordance with the Declaration of Helsinki and received ethical approval from the Ethics Committee of the ASL Lecce, Via Miglietta, n. 5, 73100 Lecce, Italy.

### Statistical Analyses

Comparison of the proportion of deceased subjects across subgroups of sex, age, and other baseline patient characteristics was performed by contingency table analysis with the Chi-square test. Kaplan–Meier product-limit survival curve estimates, and the corresponding log-rank tests, were computed to perform univariate comparisons between subgroups of baseline patient characteristics [22], according to both overall survival (OS) and relapse-free survival (RFS). Calculation of OS started from the date of initiating decitabine therapy and ended at death or at the last available date of follow-up (i.e., alive censored patients). The calculation of RFS, computed among patients who responded to treatment, started from the date of response to decitabine therapy and ended at relapse or death (i.e., events) or at the last available date of follow-up (i.e., alive censored patients). Hazard ratios (HR) of OS, and their 95% confidence intervals (CI), were estimated using multivariate Cox proportional hazards regression [23], including *a priori* defined terms for study center, sex, age, AML type (de novo vs. secondary), the presence of comorbidities, molecular biology profile, karyotype profile, blasts (>30% vs. ≤30%), haemoglobin, WBC, and platelet count at baseline. Odds ratios (OR) of response to treatment, and their 95% CI, were estimated using multivariate logistic regression, including the same covariates reported above for the Cox regression model. All tests were two-sided, and a *p*-value of less than 0.05 was considered statistically significant. Statistical analyses were performed using the SAS package, version 9.4 (SAS Institute, Cary, NC, USA), and Figures were produced using STATA software, version 15 (StataCorp, College Station, TX, USA).

## 3. Results

Patients’ characteristics at baseline and the corresponding survival data are shown in Table 1. In total, 123 males (61.8%) and 76 females (38.2%) were enrolled. The median age at diagnosis was 75.0 years (range: 61–91), and 23.1% of patients were aged 80 years or more. About half of the patients (51.0%) had ECOG PS equal to 1, and more than one-third (35.9%) had ECOG PS ≥2; only 11.1% of patients presented no concurrent diseases, while 13.6% of cases had three or more comorbidities. De novo AML was present in 92 patients (46.7%) and secondary AML in 105 patients (53.3%). As to molecular biology and karyotype profiles, an adverse classification was reported for 6.0% and 24.6% of patients, respectively. A total of 48 subjects (24.2%) had blasts ≤30%, while 150 (75.8%) had a percentage of blasts exceeding 30%. The median blood levels at baseline were 8.1 g/dL (range: 3.6–13.2) for haemoglobin, 4000 (range: 370–248,000) for WBC count, and 45,000 (range: 2000–680,000) for platelet count.

A total of 165 deaths (82.9%) for any cause occurred among the 199 patients enrolled.

**Figure 1:**
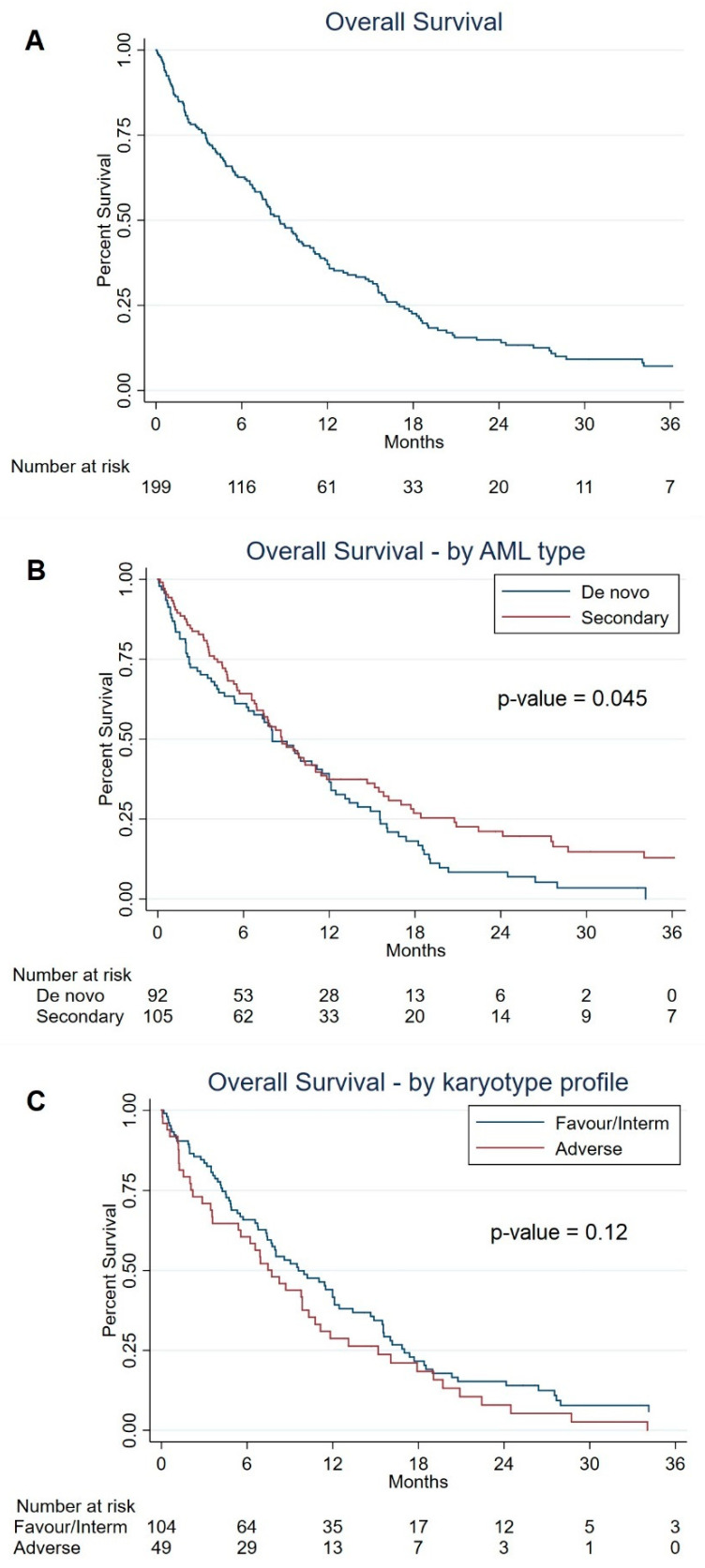
Overall survival in all patients (Panel (**A**)), in selected AML type subgroups (Panel (**B**)), and karyotype profile (Panel (**C**)). Italy, 2013–2021.

The median OS was 8.7 months (95% CI: 7.4–10.3), and the 6-month, 1-year, and 3-year OS rates were 62.7%, 37.0%, and 7.1%, respectively (Figure 1A). Regarding survival in selected subgroups of baseline patient characteristics (Table 2), the median OS was 8.0 months (95% CI: 6.2–11.5) in patients with de novo AML vs. 8.7 months (95% CI: 6.9–11.0) in those with secondary AML (univariate log-rank test, *p*-value = 0.045) (Figure 1B), and 9.6 months (95% CI: 7.4–12.1) in patients with a favorable or intermediate karyotype profile vs. 7.7 (95% CI: 3.6–10.3) in those with an adverse karyotype profile (univariate log-rank test, *p*-value = 0.12) (Figure 1C). OS according to the number of treatment cycles (<4 vs. ≥4) is provided in Supplementary Figure S1.

The multivariate HR of OS and the corresponding 95% CI, according to baseline characteristics of AML patients, are shown in Table 3. Mortality was increased in AML patients with three or more comorbidities (HR = 2.45; 95% CI: 1.18–5.08) as compared to those with no comorbidities, and in those with an adverse karyotype profile (HR = 1.58; 95% CI: 1.05–2.38) as compared to those with a favorable or intermediate profile. Furthermore, a tendency towards increased mortality emerged with higher age at diagnosis (HR = 1.02 for each 1-year increase in age; 95% CI: 0.99–1.06) and with higher WBC count (HR = 1.14 for each increase of one SD; 95% CI: 0.96–1.36), although no statistically significant relationship was found.

The best responses achieved with AML decitabine treatment are reported in Table 4. The average number of therapy cycles administered per patient was 6.3 (SD: 6.0), with a median of 5.0 cycles per patient. The maximum number of cycles administered was 35 (in one patient). CR was achieved by 31 of 199 patients (15.6%) and PR by 57 patients (28.6%), bringing to a total of 88 responders overall (44.2%). Sixty-two patients (31.2%) were non-responders, including fifty-seven (28.6%) with stable disease, and five (2.5%) with disease progression. A strong association (*p* < 0.001) emerged between the best response achieved and the number of treatment cycles (<4 vs. ≥4), as most patients achieving CR were treated with ≥4 cycles (97%, vs. 47% among non-responders).

The multivariate OR of treatment response, and the corresponding 95% CI, according to patients’ baseline characteristics, are shown in Table 5. No significant predictor of treatment response was identified, although an increased platelet count at baseline (OR = 1.88 for each increase of one SD; 95% CI: 0.99–3.58) tended to be associated with a higher probability of response to treatment.

**Figure 2:**
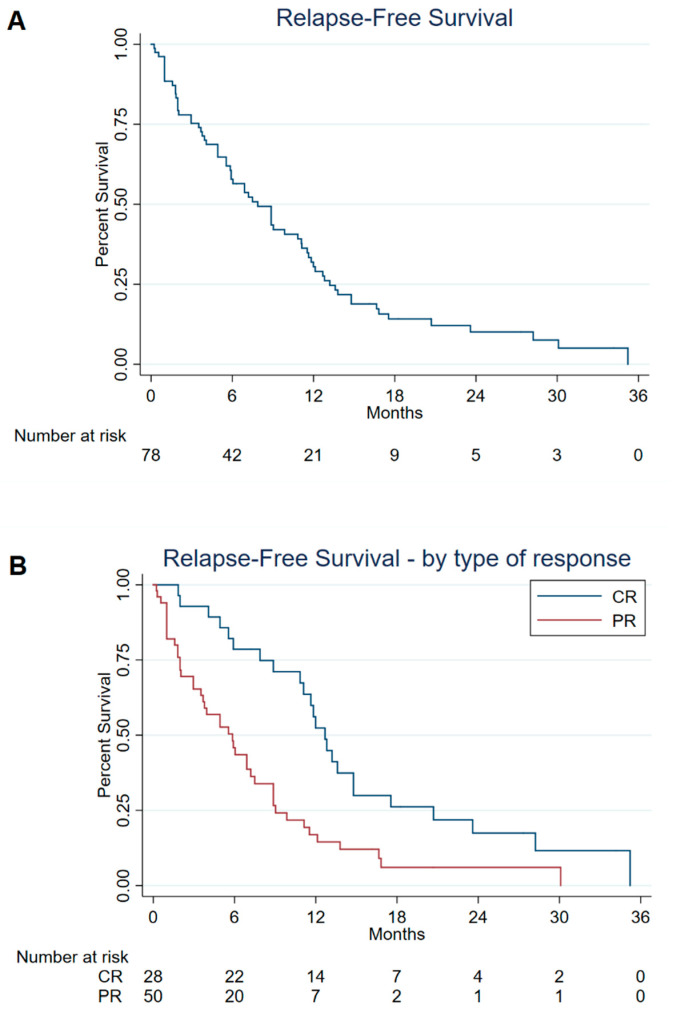
Relapse-free survival in all 78 patients who responded to treatment (Panel (**A**)) and according to type of treatment response (Panel (**B**)). ^a,b^ Italy, 2013–2021. ^a^ Including patients with complete response (CR) and partial response (PR). ^b^ Ten of 88 patients who responded to treatment could not be included in this analysis due to missing or inconsistent information on the date of achieving clinical response and/or date of disease relapse.

Figure 2 show the information on RFS in patients responding to treatment, overall (Figure 2A), and according to the type of response (Figure 2B). The median RFS was 7.9 months (95% CI: 5.8–11.1), ranging between 5.8 months (95% CI: 3.5–7.5) in patients with PR and 12.7 months (95% CI: 10.8–14.8) in those with CR (univariate log-rank test, *p*-value < 0.001).

The toxicity profile of AML patients treated with decitabine is presented in Table 6. Ninety-one patients (46.0%) suffered an infectious event, with a mean duration of fever >38° of 9.1 days (SD: 6.8). Pulmonary toxicity was observed in 11.1% of patients, gastrointestinal toxicity in 9.1%, and cardiac toxicity in 6.1% of patients. Severe toxicity (grade 3–4) due to pulmonary and cardiac events occurred in 3.1% and 2.0% of patients, respectively.

## 4. Discussion

The treatment of elderly AML patients or those unfit for intensive induction chemotherapy remains a challenge for hematologists, though the survival of these patients significantly improved with the advent of low-intensity therapeutic regimens, comprising hypomethylating agents such as decitabine or azacytidine. Here we present long-term results of the Apulian Hematologic Network real-life clinical practice study of newly diagnosed AML patients unfit for intensive chemotherapy and treated with 5-day courses of decitabine 20 mg/m^2^ daily every four weeks. Published real-world data on decitabine are scarce, are often based on small numbers of patients and/or have relatively short follow-up periods (i.e., ranging from 4 to 15 months), less than 33.6 months of the present study [9,17,18]. In addition, it is well known that older adults or those with comorbidities are under-represented in clinical trials as compared with populations of AML patients seen daily in clinical practice.

Our database included an unselected, varied set of patients. Although there were more patients aged over 70 years (85% vs. 71%), with secondary AML (53% vs. 36%), or bone marrow blasts greater than 30% (75% vs. 44%) and comorbidities (89% vs. 0%), our analysis found survival estimates and response rates comparable to those reported in the pivotal phase III randomized clinical trial of decitabine use in elderly patients with newly diagnosed AML [8]. More specifically, the median OS was 8.7 months in this analysis as compared to 7.7 months in the randomized trial, whereas CR was achieved by 15.6% and 15.7% of patients (or 17.8% when CR also included those with incomplete platelet recovery), respectively. Confirmation of these findings in real-world investigations is of utmost importance.

In 2017, a meta-analysis of clinical trials supported decitabine as a frontline treatment for AML [24]. The pooled estimates of CR, ORR, and OS were 27% (95% CI 19–36%), 37% (95% CI 28–47%), and 8.1 months (95% CI 5.8–10.4), respectively. Similar results were reported in a single-center US study, including 671 elderly AML patients newly diagnosed between 2000 and 2010 [12]. About 10% (*n* = 67) of cases were treated with decitabine, and CR was achieved by 30% of these patients (as compared to 26% for azacitidine and 42% for chemotherapy). Survival rates were similar in the epigenetic (median OS = 6.5 months) and chemotherapy (median OS = 6.7) groups, although the median OS was higher in the decitabine group (8.8 months) than in the azacitidine group (5.5 months). These results made hypomethylating agents such as azacitidine and decitabine an established standard of care for the treatment of elderly with AML. In other prior observational studies, CR ranged between 18% and 40% and the median OS was between 3.4 and about 18 months [10,11,12,14,15,17,25,26]. The difference in the CR rate observed among different studies is likely explained by different response criteria used in various analyses or by the use of a ten-day schedule of decitabine [25]. Our results from the REP study, using a prospective approach, are thus in broad agreement with previous findings.

The presence of a high number of comorbidities and an adverse karyotype profile were significant predictors of increased mortality in this AML population. In particular, the risk tended to increase with the number of concurrent diseases, from about 50% in patients with only one comorbidity (as compared to no comorbidities) to an almost 2.5-fold increased mortality rate in those with three or more comorbidities. The central role of the cytogenetic profile on mortality in AML is well known and was confirmed in our multivariate analysis [5,27]. Increasing age and higher WBC counts at baseline also showed a potential association with increased mortality in patients treated with first-line decitabine, similarly to some—but not all—previous studies [10,12,17,27]. Secondary AML has an important negative impact on survival in AML patients [8]. In our real-world series, however, no significant difference in OS emerged in multivariate analyses between de novo and secondary AML, suggesting that decitabine can overcome the negative prognostic factors observed in secondary AML with conventional chemotherapy [28]. The analysis of predictors of response to decitabine treatment, on the other hand, did not identify any clearly associated factor but only a slight tendency towards an increased response in patients with a high platelet count at baseline.

A number of adverse events occurred during the study, but decitabine was generally well tolerated, and the toxicity profile was largely consistent with earlier data. Severe cardiac, pulmonary, and gastrointestinal events were rather infrequent, i.e., they occurred in 1–3% of patients (with no fatal event registered), while no severe hepatic, genitourinary, and metabolic events occurred. Infectious events were frequently reported (in 46% of cases), leading on average to 9.1 days of fever requiring hospitalization. In the already cited meta-analysis of clinical trials on decitabine use in elderly AML patients [24], pneumonia (25%) and sepsis (9%) were the most frequent infectious complications. No information was, however, collected on the type of infectious events in our study. Given the good safety outline of decitabine and some limits observed in its duration of response [18], several clinical trials have investigated the combination of decitabine with other agents in AML [29]. DiNardo et al. recently reported the results of a single centre, phase II study with decitabine and venetoclax in 75 elderly patients with newly diagnosed or untreated secondary AML. The overall response rate was 89% in newly diagnosed AML and 80% in untreated secondary AML, while the median overall survival was 18.1 months in newly diagnosed AML and 7.8 months in untreated secondary AML. The most common treatment-emergent adverse events included infections with grades 3 or 4 neutropenia in 47% of cases [30]. A recently published large randomized, phase III study comparing azacitidine plus either venetoclax or placebo in previously untreated AML patients ineligible for standard induction therapy reported a higher rate of complete remission or complete remission with incomplete hematologic recovery with azacitidine–venetoclax than with the control regimen (66.4% vs. 28.3%) as well as better median overall survival (14.7 vs. 9.6 months). Infections of any grade occurred in 84% of the patients in the azacitidine—venetoclax group and 67% in the control group [31]. At the time of initiation of this study, decitabine alone was an established first-line regimen in AML patients.

Promising results on the effectiveness of combination therapy, with a high response rate in the absence of significant toxicity drawbacks, were also recently reported by our working group in a preliminary real-world analysis of 56 patients with high-risk AML treated with decitabine plus venetoclax, achieving a complete response rate (CR + CRi) of 60% after a median of two cycles [32].

The limitations of this analysis are those typical of observational studies. Errors in reporting the patients’ characteristics, particularly self-reported information at baseline (e.g., presence of comorbidities), cannot be excluded. Still, most variables examined in multivariate models were of a clinical nature or based on laboratory assessments, thus reducing the risk of information bias. Furthermore, the registration of adverse events in observational studies lacks the rigorousness of clinical trials thus toxicity effects may be somewhat underestimated in this analysis. On the other hand, selection bias should not be a major issue, as all consecutive AML patients presenting to the participating study centers and complying with the inclusion criteria were enrolled. The strengths of this study are its long follow-up period (a median of almost 3 years, calculated using the method proposed by Schemper and Smith [20]), the relatively large sample size allowing adjustment for several baseline covariates, and the inclusion of a varied set of frail, comorbid, or high-risk patients, since 23% of cases were aged ≥80 years, over one-third had ECOG PS ≥2 and more than half presented with secondary AML. Of note, when we further examined the relationship between the number of patient comorbidities and early treatment discontinuation (i.e., less than four cycles received), no association was found (data not shown). This indicates that decitabine is well tolerated in unfit patients, and may suggest a favorable role of decitabine in disease-related fitness dynamics during treatment [33].

## 5. Conclusions

We provided results from the Apulian Hematologic Network obtained with a prospective real-world study, supporting previous indications on safety and effectiveness, and further quantifying the favorable role of first-line decitabine use in the treatment of AML in clinical practice, suggesting that it may be a good companion for new therapeutic combinations for the treatment of this challenging disease.

## Figures and Tables

**Table 1:** Patients’ characteristics at baseline. Italy, 2013–2021.

Characteristic	No. Patients (%)
Sex	
Females	76 (38.2)
Males	123 (61.8)
Age at diagnosis (years)	
<70	30 (15.1)
70–74	61 (30.6)
75–79	62 (31.2)
≥80	46 (23.1)
Median (range)	75.0 (61–91)
ECOG PS	
0	26 (13.1)
1	101 (51.0)
≥2	71 (35.9)
Missing, n	1
Type of AML	
De novo	92 (46.7)
Secondary	105 (53.3)
Missing, n	2
Comorbidities	
No	22 (11.1)
1	90 (45.2)
2	60 (30.1)
≥3	27 (13.6)
Molecular biology profile	
Favorable	11 (5.5)
Intermediate	103 (51.8)
Adverse	12 (6.0)
Not available	73 (36.7)
Karyotype profile ^a^	
Favorable/Intermediate	104 (52.3)
Adverse	49 (24.6)
Not available	46 (23.1)
Blasts (%)	
≤30	48 (24.2)
>30	150 (75.8)
Missing, n	1
Haemoglobin (g/dL)	
Median (range)	8.1 (3.6–13.2)
WBC count	
Median (range)	4000 (370–248,000)
Platelets count	
Median (range)	45,000 (2000–680,000)
Total patients	199

AML: acute myeloid leukemia; WBC: white blood cell. ^a^ Only 5 (2.5%) patients had a favourable karyotype profile thus favorable and intermediate profiles were grouped together.

**Table 2:** Median OS according to patients’ characteristics at baseline. Italy, 2013–2021.

Characteristic	Median OS (95% CI)	*p*-Value ^a^
Sex		
Females	9.9 (6.6–12.0)	
Males	8.0 (6.8–10.3)	0.75
Age at diagnosis (years)		
<70	15.5 (8.6–20.8)	
70–74	7.5 (4.9–11.0)	
75–79	8.6 (6.6–10.8)	
≥80	9.5 (3.5–12.5)	0.16
ECOG PS		
0	11.0 (6.6–18.4)	
1	7.7 (5.4–9.5)	
≥2	10.3 (6.9–12.1)	0.19
Type of AML		
De novo	8.0 (6.2-11.5)	
Secondary	8.7 (6.9-11.0)	**0.045**
Comorbidities		
No	11.0 (6.6–19.7)	
1	9.5 (6.2–11.8)	
2	8.0 (5.4–12.0)	
≥3	7.4 (4.7–10.8)	0.65
Molecular biology profile		
Favorable	9.0 (3.5–94.0)	
Intermediate	9.6 (7.5–12.1)	
Adverse	5.5 (0.7–19.0)	0.44
Not available	7.8 (4.9–9.9)	
Karyotype profile ^b^		
Favorable/Intermediate	9.6 (7.4–12.1)	
Adverse	7.7 (3.6–10.3)	0.12
Not available	8.6 (3.2–14.0)	
Blasts (%)		
≤30	10.3 (7.4–12.1)	
>30	8.0 (6.6–9.9)	0.34
Haemoglobin (g/dL) ^c^		
<7.5	10.3 (7.7–15.2)	
7.5–<8.2	6.8 (3.6–9.9)	
8.2–<9	7.4 (3.5–12.1)	
≥9	9.8 (6.9–15.5)	0.09
WBC count ^c^		
<1810	8.2 (6.9–12.5)	
1810–<4000	8.6 (5.6–11.8)	
4000–<19,210	9.0 (5.3–14.7)	
≥19,210	7.7 (4.8–11.5)	0.87
Platelet count ^c^		
<21,000	7.7 (4.3–13.1)	
21,000–<45,000	8.0 (4.8–12.0)	
45,000–<89,000	7.5 (4.9–11.0)	
≥89,000	10.0 (8.0–15.6)	0.87
Total patients	8.7 (7.4–10.3)	

AML: acute myeloid leukemia; CI: confidence interval; OS: overall survival; WBC: white blood cell. Statistically significant results are reported in bold. ^a^ Log-rank tests. “Not available” category, not included in the calculation. ^b^ Only 5 (2.5%) patients had a favourable karyotype profile thus favorable and intermediate profiles were grouped together. ^c^ Distribution according to approximate quartiles.

**Table 3:** Multivariate hazard ratios of overall survival, and 95% confidence intervals, according to selected characteristics of patients at baseline. Italy, 2013–2021.

Patient Characteristic	HR (95% CI) ^a^
Sex (reference: females)	
Males	1.15 (0.80–1.65)
Age, 1 year increase	1.02 (0.99–1.06)
AML type (reference: De novo AML)	
Secondary AML	0.81 (0.55–1.18)
Comorbidities (reference: no)	
1	1.53 (0.82–2.83)
2	1.63 (0.86–3.06)
≥3	**2.45 (1.18–5.08)**
Molecular biology profile (reference: favourable)	
Intermediate	1.22 (0.52–2.87)
Adverse	2.00 (0.69–5.80)
Karyotype profile (reference: favourable/intermediate)	
Adverse	**1.58 (1.05–2.38)**
Blasts (reference: ≤30%)	
>30%	1.10 (0.72–1.67)
Haemoglobin, increase equal to 1 SD	0.89 (0.74–1.08)
WBC count, increase equal to 1 SD	1.14 (0.96–1.36)
Platelet count, increase equal to 1 SD	1.05 (0.86–1.30)

AML: acute myeloid leukemia; CI: confidence interval; HR: hazard ratio; SD: standard deviation; WBC: white blood cells. Statistically significant results are reported in bold. ^a^ HRs from multivariate Cox regression models, including terms for study center, sex, age, AML type, presence of comorbidities, molecular biology profile, karyotype profile, blasts, haemoglobin, WBC, and platelet count at baseline.

**Table 4:** Best response achieved with AML treatment. Italy, 2013–2021.

Best Response	No. (%)
Complete response	31 (15.6%)
Partial response	57 (28.6%)
Total responders	88 (44.2%)
Stable disease	57 (28.6%)
Disease progression	5 (2.5%)
Total non-responders	62 (31.2%)
Not evaluable ^a^	49 (24.6%)

^a^ Including 11 patients with hematological improvement and 38 patients considered “not evaluable” by clinicians (31 of them had undergone less than 4 therapy cycles).

**Table 5:** Predictors of response to treatment. Multivariate odds ratios of response and corresponding 95% confidence intervals, according to selected covariates in 150 AML patients ^a^.

Covariate	Multivariate OR ^b^ (95% CI)
Sex (reference: females)	
Males	0.64 (0.29–1.42)
Age, 1 year increase	0.94 (0.86–1.02)
AML type (reference: De novo AML)	
Secondary AML	1.08 (0.45–2.58)
Comorbidities (reference: no)	
Yes	1.20 (0.33–4.41)
Molecular biology profile (reference: favorable)	
Intermediate	4.53 (0.97–21.29)
Adverse	1.76 (0.22–14.00)
Karyotype profile (reference: favorable/intermediate)	
Adverse	1.00 (0.36–2.79)
Blasts (reference: ≤30%)	
>30%	1.77 (0.67–4.62)
Haemoglobin, increase equal to 1 SD	0.80 (0.52–1.24)
WBC count, increase equal to 1 SD	0.93 (0.54–1.59)
Platelet count, increase equal to 1 SD	1.88 (0.99–3.58)

AML: acute myeloid leukemia; CI: confidence interval; OR: odds ratio; WBC: white blood cell. ^a^ A total of 49 patients with non-evaluable responses were excluded from this analysis. ^b^ ORs from multiple logistic regression models, including terms for study center, sex, age, AML type, presence of comorbidities, molecular biology profile, karyotype profile, blasts, haemoglobin, WBC, and platelet count at baseline.

**Table 6:** Toxicity profile in 199 AML patients treated with decitabine. Italy, 2013–2021 ^a,b^.

Type of Toxicity	No. (%)
Cardiac toxicity	
Any	12 (6.1)
Severe	4 (2.0)
Pulmonary toxicity	
Any	22 (11.1)
Severe	6 (3.1)
Genitourinary toxicity	
Any	5 (2.5)
Severe	0 (0.0)
Hepatic toxicity	
Any	6 (3.0)
Severe	0 (0.0)
Gastrointestinal toxicity	
Any	18 (9.1)
Severe	2 (1.1)
Metabolic toxicity	
Any	6 (3.0)
Severe	0 (0.0)
Infectious events	
Any	91 (46.0)
Days with fever >38°, mean ± SD ^c^	9.1 ± 6.8

^a^ The percentages take into account the presence of some missing information. ^b^ Severe events: grade 3–4. ^c^ Computed in patients with infectious events only.

## Data Availability

The data presented in this study are available on reasonable request from the corresponding author. The data are not publicly available due to privacy restrictions.

## Supplementary Materials

The following supporting information can be downloaded at: https://www.mdpi.com/article/10.3390/cancers14030826/s1, Figure S1: Overall survival according to the number of treatment cycles (<4 vs. ≥4). Italy, 2013–2021.
