# Supplementary figures and images for: Long-Term Follow-Up of Elderly Patients with Acute Myeloid Leukemia Treated with Decitabine: A Real-World Study of the Apulian Hematological Network
Source: Cancers (Basel). 2022 Feb 6;14(3):826. doi: 10.3390/cancers14030826 (PMC8834602; doi:10.3390/cancers14030826)

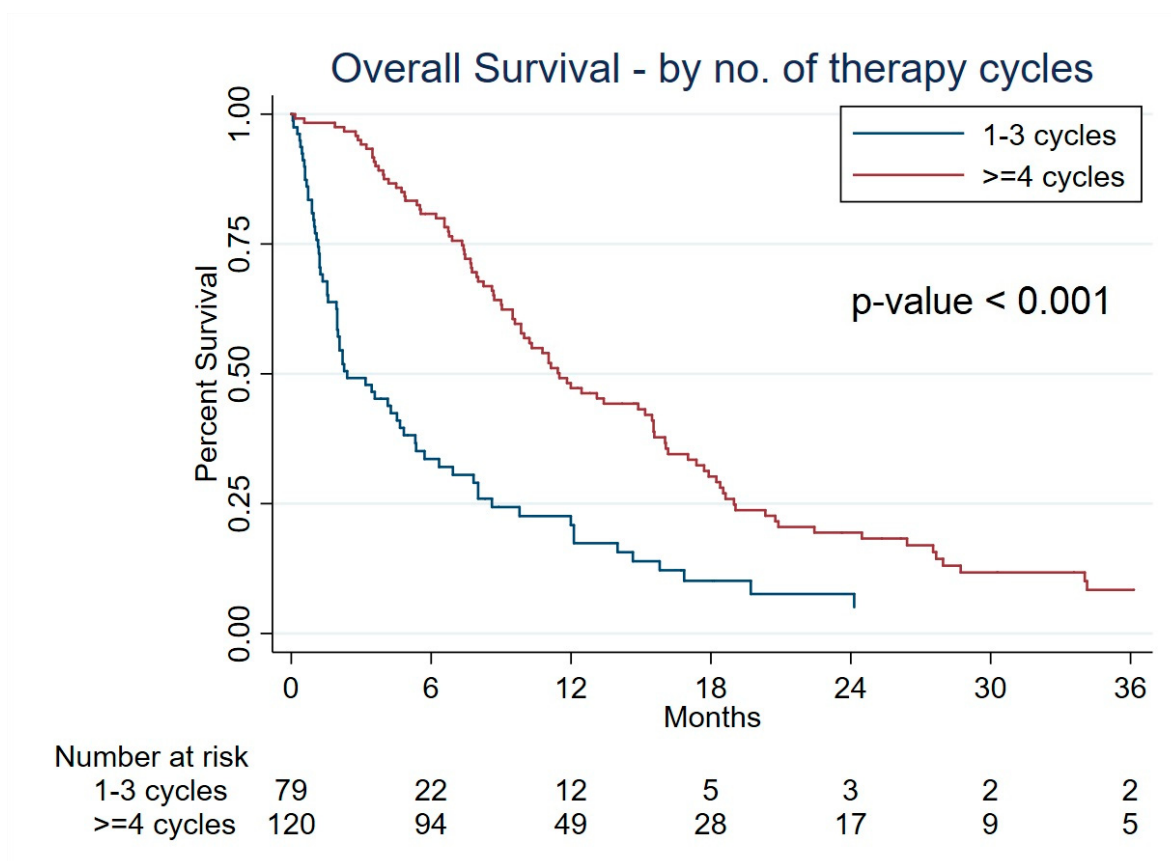

**Figure S1.** Overall survival according to the number of treatment cycles (<4 vs. ≥4). Italy, 2013-2021.

Supplement: Supplementary file 1 [file cancers-14-00826-s001.zip › cancers-1575893-supplementary.pdf]
